# Effects of goal-directed fluid management guided by a non-invasive device on the incidence of postoperative complications in neurosurgery: a pilot and feasibility randomized controlled trial

**DOI:** 10.1186/s13741-023-00321-3

**Published:** 2023-07-05

**Authors:** Ondrej Hrdy, Milos Duba, Andrea Dolezelova, Ivana Roskova, Martin Hlavaty, Rudolf Traj, Vit Bönisch, Martin Smrcka, Roman Gal

**Affiliations:** 1 Department of Anesthesiology and Intensive Care Medicine, University Hospital Brno, Brno, Czech Republic; 2 Department of Anesthesiology and Intensive Care Medicine, Faculty of Medicine, Masaryk University, Brno, Czech Republic; 3 Department of Neurosurgery, University Hospital Brno, Brno, Czech Republic; 4 Department of Neurosurgery, Faculty of Medicine, Masaryk University, Brno, Czech Republic

**Keywords:** Complications, Goal-directed therapy, Hemodynamic monitoring, Neuro-anesthesia, Neurosurgery, Postoperative outcome

## Abstract

**Background:**

The positive effects of goal-directed hemodynamic therapy (GDHT) on patient-orientated outcomes have been demonstrated in various clinical scenarios; however, the effects of fluid management in neurosurgery remain unclear. Therefore, this study was aimed at assessing the safety and feasibility of GDHT using non-invasive hemodynamic monitoring in elective neurosurgery. The incidence of postoperative complications was compared between GDHT and control groups.

**Methods:**

We conducted a single-center randomized pilot study with an enrollment target of 34 adult patients scheduled for elective neurosurgery. We randomly assigned the patients equally into control and GDHT groups. The control group received standard therapy during surgery and postoperatively, whereas the GDHT group received therapy guided by an algorithm based on non-invasive hemodynamic monitoring. In the GDHT group, we aimed to achieve and sustain an optimal cardiac index by using non-invasive hemodynamic monitoring and bolus administration of colloids and vasoactive drugs. The number of patients with adverse events, feasibility criteria, perioperative parameters, and incidence of postoperative complications was compared between groups.

**Results:**

We successfully achieved all feasibility criteria. The GDHT protocol was safe, because no patients in either group had unsatisfactory brain tissue relaxation after surgery or brain edema requiring therapy during surgery or 24 h after surgery. Major complications occurred in two (11.8%) patients in the GDHT group and six (35.3%) patients in the control group (*p* = 0.105).

**Conclusions:**

Our results suggested that a large randomized trial evaluating the effects of GDHT on the incidence of postoperative complications in elective neurosurgery should be safe and feasible. The rate of postoperative complications was comparable between groups.

**Trial registration:**

Trial registration: ClininalTrials.gov, registration number: NCT04754295, date of registration: February 15, 2021.

**Supplementary Information:**

The online version contains supplementary material available at 10.1186/s13741-023-00321-3.

## Background

Surgical procedures, like other treatments, carry a risk of complications and unsatisfactory clinical outcomes, such as infections, seizures, thromboembolic events, prolonged ICU and hospital lengths of stay, and mortality (Tevis et al., 2013; Dencker et al. 2021). Goal-directed hemodynamic therapy (GDHT) is currently recommended in several surgical subspecialties to decrease the incidence of perioperative complications (Gustafsson et al. 2019; Wainwright et al. 2020; Batchelor et al. 2019). GDHT also decreases the incidence of postoperative complications, such as thromboembolic events and wound infections. In addition, GDHT decreases hospital lengths of stay and costs in orthopedic surgery (Calvo-Vecino et al. 2018; Salzwedel et al. 2013; Benes et al., 2015) and major abdominal surgery and has been associated with improved outcomes in major surgery (Hamilton et al. 2011; Benes et al. 2014; Gan et al. 2002; Miller et al. 2014).

GDHT involves optimizing patients’ hemodynamics by using advanced monitoring systems, which enable the determination of several hemodynamic parameters, notably cardiac output and the cardiac index (CI).

Adequate cardiac output is essential for sufficient oxygen delivery to tissues, to avoid tissue hypoxia. Optimal preloading is mandatory to achieve sufficient cardiac output (Vincent et al., 2013).

The intravascular volume may be insufficient in patients undergoing neurosurgical operations for several reasons. These patients often receive diuretics before surgery for the treatment of cerebral edema; oral intake may decrease, and patients may be at risk of blood loss. Diminished blood flow and oxygen delivery due to hypovolemia place patients at risk of organ dysfunction (Bellamy 2006). However, fluid overload may compromise cardiac output and can also aggravate cerebral edema. Thus, appropriate fluid management is a challenge during neurosurgical procedures. Various parameters can be used to guide fluid and hemodynamic management. Measurement of static or dynamic parameters can be used. However, static parameters, such as central venous pressure or mean arterial pressure (MAP), despite their frequent use, are poor predictors of fluid responsiveness (Cavallaro et al. 2008; Marik et al., 2008). Dynamic parameters, such as stroke volume variation, have been shown to sensitively predict fluid responsiveness (Hofer et al. 2008; Vos et al. 2015). Although positive effects on patient-orientated outcomes have been demonstrated in major surgery, knowledge regarding the effects of fluid management in neurosurgery remains limited (Luo et al. 2017; Wu et al. 2017). Various devices can be used to assess patients’ hemodynamic status. Most such devices are invasive and require an arterial line to be in place. Non-invasive devices are also available, such as the Starling SV monitor, a completely non-invasive hemodynamic monitor using patented Bioreactance® technology to monitor patients’ hemodynamic status. However, only limited data on its use in neurosurgery have been reported (Sivakumar et al., 2020).

The aim of a feasibility study is to assess whether a future large trial can be performed (Eldridge et al. 2016). We conducted the current study to determine the feasibility, safety, and efficacy of GDHT using the non-invasive Starling SV hemodynamic monitor in elective neurosurgery and to obtain data to aid in planning a future fully powered randomized clinical trial.

## Methods

In this single-center randomized controlled trial, we aimed to enroll 34 adult patients undergoing elective neurosurgery. The Ethical Committee of University Hospital Brno approved this study (Brno, Czech Republic; February 17, 2021), and the study was registered at ClinicalTrials.gov before patient enrollment (NCT04754295, principal investigator: R. Gal, date of registration: February 15, 2021). This study was performed at the Department of Anesthesiology and Intensive Care and the Department of Neurosurgery of University Hospital Brno and Masaryk University in Brno between March 2021 and September 2021. The study was performed and is reported in accordance with the applicable Consolidated Standards of Reporting Trials (CONSORT) statement (Schultz et al., 2010).

All patients admitted to the Department of Neurosurgery were screened according to the following inclusion criteria: (i) age ≥ 18 years, (ii) expected duration of surgery ≥ 2 h, (iii) provision of written informed consent, and (iv) American Society of Anesthesiologists (ASA) categories 1–3. Patients were excluded if they met any of the following exclusion criteria: (i) ASA category IV, (ii) surgery for traumatic brain injury or acute hemorrhagic stroke, (iii) osmotherapy before surgery, (iv) operative position other than lateral or supine, (v) awake neurosurgery, (vi) unavailability of hemodynamic monitoring data, or (vii) cardiac arrhythmia. Written informed consent was obtained from all patients before enrollment.

Participants were randomly allocated in a 1:1 ratio to the two study arms, according to a sequence generated by a computer. MedCalc Statistical Software version 19.3.1 (MedCalc Software Ltd., Ostend, Belgium; https://www.medcalc.org; 2020) was used to generate the randomization list. Subsequently, 34 sequentially numbered opaque-sealed envelopes were prepared according to the randomization list, each containing a note indicating the study arm (GDHT or STANDARD). The envelopes were sealed by an independent member of the study team. Randomization was performed after patient recruitment by a study team member shortly before the surgical procedure at the operating theater. Blinding was applied to only the attending neurosurgeons, to ensure that the evaluation of brain tissue relaxation was not affected by knowledge of patient allocation. The hemodynamic monitor was in place in all patients but was actually used only in the GDHT group. The monitor screen was not visible to the neurosurgeons, who therefore could not determine whether the device was being used. Because of the nature of the trial interventions, blinding of other staff in the operation theater and other study members was not possible. The data management group and statisticians processed a pseudo-anonymized data set.

### Management of general anesthesia

General anesthesia was induced by intravenous administration of propofol (2 mg/kg). For attenuation of the hemodynamic response to laryngoscopy, sufentanil (0.1 mcg/kg) was added. To facilitate intubation, rocuronium (0.6 mg/kg) was used. After tracheal intubation, artificial ventilation was commenced with the following settings: tidal volume of 8–10 ml/kg and respiratory rate of 10–14 breaths/min, to maintain an end-tidal CO_2_ of 30–35 mmHg. Anesthesia was maintained with propofol infusion (4–12 mg/kg/h), and additional doses of opioids and relaxants were administered at the discretion of the attending anesthesiologist. The patients’ temperature was maintained at 36–37 °C with a circulating water blanket.

### Hemodynamic monitoring and management

Both groups received standard monitoring of vital signs through continuous electrocardiography, pulse oximetry, end-tidal CO_2_ measurement, and invasive monitoring of arterial blood pressure. In the STANDARD arm, perioperative hemodynamic management and postoperative care were at the discretion of the attending anesthesiologist and intensivist, respectively. Hypotension with *MAP* < 65 mmHg was managed with administration of a bolus of 500-l crystalloid over 10 min (Isolyte, Fresenius Kabi Deutschland GmbH, 61169 Friedberg, Germany) or 250-ml colloid over 5 min (Gelaspan 4%, B. Braun Melsungen AG, 34212 Melsungen, Germany). The choice of fluid and number of boluses were at the discretion of attending anesthesiologist. If fluids were considered ineffective, a vasopressor was added. In the GDHT arm, non-invasive hemodynamic monitoring was used (Starling™ SV hemodynamic monitor, Cheetah Medical Inc., 600SE Maritime Ave Suite 220, Vancouver, WA, USA) in addition to the monitoring used in the STANDARD arm. The Starling SV monitor uses patented Bioreactance® technology and requires placement of only four sensors, which are skin electrodes similar to those used for ECG monitoring. The extended monitoring began after the induction of general anesthesia and resolution of any hypotension caused by the induction of general anesthesia. This hypotension was resolved with fluid bolus and vasopressor administration if necessary. Subsequently, a basal infusion was initiated with balanced crystalloid solution (Isolyte, Fresenius Kabi Deutschland GmbH, 61169 Friedberg, Germany) at 3 ml · kg^−1^ · h^−1^ with an infusion pump. We then determined the optimal CI, defined by a value of at least 2.5 l · min · m^−2^, a stroke volume variation (SVV) below 15%, and a MAP above 65 mmHg. If the CI was not optimal, appropriate interventions were performed to achieve optimal values. In the case of SVV > 15%, a bolus of 250 ml of colloid was administered over 5 min (Gelaspan 4%, B. Braun Melsungen AG, 34212 Melsungen, Germany). This process was repeated until the SVV decreased below 15%. If the SVV was above 15%, and the CI decreased with administration of a bolus of colloid, inotropic therapy was introduced (dobutamine) at a dose of 2.5 mcg/kg/min and increased if necessary to achieve a *CI* > 2.5 l · min · m^−2^. If hypotension with MAP below 65 mmHg and an SVV below 15% occurred, norepinephrine therapy was started.

After determination of optimal CI, we aimed to maintain the SVV below 15% to preserve the optimal CI. When the SVV exceeded 15%, and the CI was lower than optimal, a colloid (Gelaspan 4%, B. Braun Melsungen AG, 34212 Melsungen, Germany) bolus (250 ml) was administered. If, after administration of colloid, the SVV decreased below 15%, the CI was verified. If the CI was optimal, no other action was taken. If the CI remained suboptimal, the colloid bolus administration was repeated. If the SVV remained above 15% despite administration of the colloid bolus, the CI was verified. If the CI increased, no other action was taken. If the CI was suboptimal, inotropic therapy was introduced (dobutamine). In the event of hypotension with MAP below 65 mmHg concurrent with an SVV above 15%, a 250-ml colloid bolus was administered. If hypotension with MAP below 65 mmHg and an SVV below 15% occurred, norepinephrine therapy was started. The hemodynamic management is summarized in Supplementary Fig. 1 (Additional file 1).

### Study end points

Feasibility end points included less than 55% of potentially eligible patients missed; consent rate > 75%; recruitment of 34 patients within 6 months; < 10% missing outcome data, including ICU and hospital length of stay (LOS), and survival; and < 10% missing clinical data obtained from clinical medical notes and electronic patient records. Safety end points included the number of patients with adverse events (AE), defined as unsatisfactory brain tissue relaxation at the end of surgery and brain edema requiring therapy during surgery or 24 h after surgery. The incidence of AE was compared between the control and intervention groups. Brain tissue relaxation was evaluated by a neurosurgeon after incision of the dura and at the end of the surgery just before suturing of the dura. Brain tissue relaxation was classified according to four categories: 1, fully relaxed; 2, sufficiently relaxed; 3, stiff; and 4, bulging. Categories 3 and 4 were considered unsatisfactory (Li et al. 2016). Brain edema requiring therapy was defined by any change in patient condition considered to be associated with the development of, or deterioration due to, brain edema requiring any pharmacological or non-pharmacological therapy. The efficacy end points compared between groups were the amounts of infused crystalloids, colloids, transfusions, and blood loss during surgery, the number of patients with hypotensive episodes (*MAP* < 65 torr), and the number of patients with vasoactive drug intervention. We also measured urine output during surgery and 24 h postoperatively in both groups. In the 24-h postoperative period, we measured the amounts of administered crystalloids and colloids. We assessed the numbers of patients requiring transfusion and the number of transfusion units administered in the 24-h postoperative period. In both groups, we quantified serum hemoglobin and lactate levels 24 h after surgery and compared these values between groups.

The incidence of postoperative complications during the hospital stay was compared between groups. A list and definitions of complications is provided in Supplementary Table 1 (Additional file 2). The following clinical outcomes were compared between groups: (i) hospital LOS, with the day of admission and day of discharge counted as 1 day; (ii) ICU LOS, with the day of admission and day of discharge counted as 1 day; and (iii) 28-day mortality.

### Statistical analysis

Because this was a pilot study, a power calculation to determine sample size was not performed. We aimed to achieve a final sample size of 30 participants, according to standard guidelines for pilot studies (Lancaster et al. 2004). Expecting a 10% dropout rate, we sought to recruit a total of 34 participants. Normally distributed data are presented as mean ± standard deviation. Categorical data are presented as numbers and percentages. The primary outcome (incidence of AE) was compared between groups with Fisher’s exact test. The secondary outcomes were compared between groups with independent sample Student’s *t*-tests for normally distributed continuous data or Pearson’s chi-square test for categorical data. All statistical tests were two-sided, and *p*-values < 0.05 were considered to indicate significant differences. We adhered to the intention-to-treat principle. The statistical analyses were performed in MedCalc Statistical Software version 19.3.1 (MedCalc Software Ltd., Ostend, Belgium; https://www.medcalc.org; 2020).

## Results

This prospective randomized controlled pilot trial was performed at the.

**Fig. 1:**
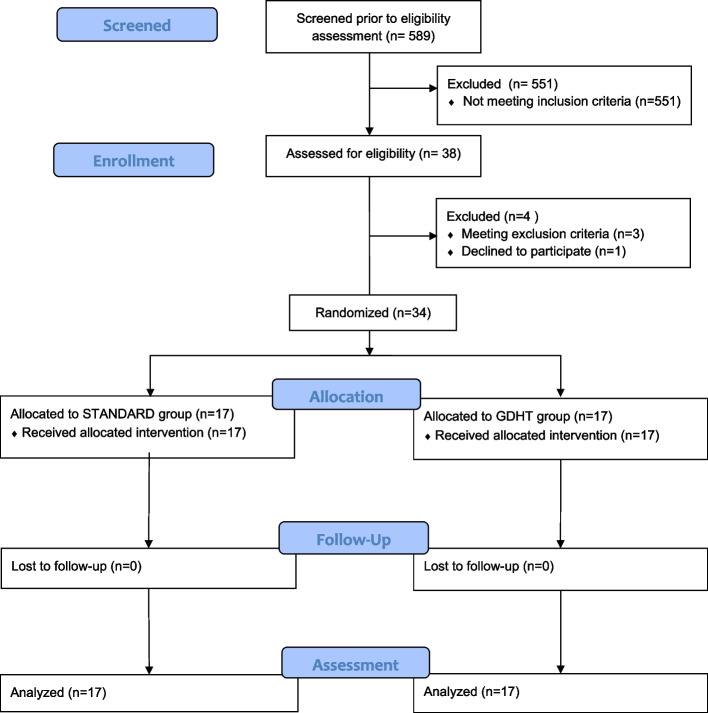
CONSORT flow diagram. CONSORT indicates Consolidated Standards of Reporting Trials

Department of Anesthesiology and Intensive Care and Department of Neurosurgery of University Hospital Brno and Masaryk University in Brno between March 2021 and September 2021. A total of 34 patients were included and randomized in this study. No patients dropped out, and data for all randomized patients were included in the final analysis (Fig. 1).

### Baseline data

The patient characteristics are summarized in Table 1. No statistically significant differences in age, sex, and ASA category were identified between groups. The feasibility outcome measures are summarized in Table 2. All criteria assessing feasibility were met. Ninety-seven percent of eligible patients agreed to participate, and 34 patients were enrolled during 6 months. The demographic characteristics were balanced (Table 1), and no data from the patients’ medical records were missing.

**Table 1:** Patient characteristics

Variable	GDHT (*n* = 17)	STANDARD (*n* = 17)	*p*
Age, y	56.9 ± 16.8	58.4 ± 9.5	0.881
Sex			0.398
Male	2 (11.8)	5 (29.4)	
Female	15 (88.2)	12 (70.6)	
ASA			0.779
I	1 (5.9)	1 (5.9)	
II	9 (52.9)	7 (41.2)	
III	7 (41.2)	9 (52.9)	
Comorbidities			
Arterial hypertension	10 (58.8)	11 (64.7)	0.724
Ischemic heart disease	3 (17.6)	2 (11.8)	0.628
Diabetes mellitus	4 (23.5)	2 (11.8)	0.358
Severe pulmonary disease	0	0	-
Indication for surgery			1.0
Primary brain tumor	17	16	
Metastastic tumor	0	1	

Continuous data are expressed as mean ± standard deviation. Categorical data are expressed as the number of patients (percentage)

*Abbreviations*: *ASA* American Society of Anaesthesiologists

**Table 2:** Feasibility criteria assesment

Trial process	Outcome measure	Feasibility criteria	Value *n* (%)	Criterion fulfilled (yes/no)
Screening	Percentage of potentially eligible patients missed	< 55% of potentially eligible patients missed	0	Yes
Consent	Percentage of eligible participants agreeing to enrolment	> 75% participants agreeing to enrolment	34 (97)	Yes
Recruitment rate	Number of patients recruited	Recruit 34 patients within 7 months	34 within 7 months	Yes
Randomization	Demographic characteristics in the intervention and control arms	Balanced demographic characteristics in intervention and control arm participants	See Table 1	Yes
Electronic case report form data collection	Percentage of missing outcome and clinical data	< 10% missing outcome data including ICU and hospital length of stay and survival	0	Yes
		< 10% missing clinical data obtained from clinical medical notes and electronic patient records	0	Yes

### Safety

The safety outcome measures (number of patients with unsatisfactory brain tissue relaxation at the end of surgery and number of patients with brain edema requiring therapy during surgery or 24 h after surgery) were comparable between groups (Table 3). In addition, the number of patients with fluid bolus administration during the surgery and the number of patients with vasoactive drug administration were comparable between groups (*p* = 0.086 and *p* = 0.271, respectively).

**Table 3:** Perioperative data

Variable	GDHT (*n* = 17)	STANDARD (*n* = 17)	*p*-value
**Beginning of surgery**			
MAP, mmHg	106 ± 13	109 ± 17	0.478
Heart rate, beats/min	71 ± 11	66 ± 12	0.294
Hb, g/L	137 ± 11	135 ± 18	0.826
Patients with unsatisfactory brain tissue relaxation	4 (23.5)	4 (23.5)	1
**During surgery**			
Patients transfused	3 (17.6)	2 (11.8)	0.628
Patients with hypotension episode	7 (41.2)	6 (35.3)	0.724
Patients with vasoactive drug intervention	7 (41.2)	4 (23.5)	0.271
Patients with fluid bolus	11 (64.7)	6 (35.3)	0.086
Patients with brain edema requiring intervention	0	0	-
**End of surgery**			
MAP, mmHg	81 ± 11.8	81 ± 11.5	0.928
Heart rate, beats/min	61 ± 10.35	61 ± 13.49	0.984
Hb, g/l	108 ± 13.75	110 ± 16.87	0.810
Lactate, mmol/l	1.5 ± 0.92	2.1 ± 0.92	0.407
Patients with unsatisfactory brain tissue relaxation	0	0	-
Blood loss, ml	279 ± 305.19	316 ± 234.68	0.271
Crystalloids, ml	1288 ± 591.22	1935 ± 648.98	0.001
Colloids, ml	471 ± 522.03	88 ± 196.48	0.015
Duration of surgery, min	237 ± 85.92	223 ± 82.50	0.667
**24 h after surgery**			
Blood loss, ml	88 ± 85.62	75 ± 48.80	0.920
Urine output, ml	2041 ± 605.50	2191 ± 775.45	0.603
Hb, g/l	115 ± 10.58	116 ± 10.96	0.881
Lactate, mmol/l	1.8 ± 0.70	1.8 ± 0.70	0.719
Crystalloids, ml	1221 ± 507.46	1309 ± 500.70	0.490
Patients transfused	0	0	-
Patients with brain edema requiring intervention	0	0	-

Continuous data are expressed as mean ± standard deviation. Categorical data are expressed as the number of patients (percentage)

*Abbreviations*: *ASA* American Society of Anesthesiologists, *MAP* Mean arterial pressure, *Hb* Hemoglobin

### Efficacy

The outcome measures and postoperative complications are shown in Table 4. The number of patients with any major or minor complication was comparable between groups (*p* = 0.105 and *p* = 0.545, respectively). The ICU LOS and hospital LOS were also similar between groups (*p* = 0.569 and *p* = 0.976, respectively).

**Table 4:** Postoperative complications and outcomes

Complication/outcome	GDHT (*n* = 17)	Standard (*n* = 17)	*p*-value
Cardiovascular			
Minor	11 (64.7)	12 (70.6)	0.714
Major	0 (0.0)	1 (5.9)	1
Pulmonary			
Minor	10 (58.8)	11 (64.7)	0.724
Major	1 (5.9)	3 (17.6)	0.287
Infection			
Minor	1 (5.9)	4 (23.5)	0.146
Major	0	0	-
Renal			
Minor	0	0	-
Major	0	0	-
Gastrointestinal			
Minor	7 (42.2)	7 (42.2)	1
Major	0	0	-
Coagulation			
Minor	1 (5.9)	2 (11.8)	0.545
Major	2 (11.8)	3 (17.6)	0.628
Neurological			
Minor	4 (23.5)	2 (11.8)	0.628
Major	0 (0.0)	0 (0.0)	-
Any minor complication	16 (94.1)	15 (88.2)	0.545
Any major complication	2 (11.8)	6 (35.3)	0.105
Any complications	15 (88.2)	16 (94.1)	0.545
ICU LOS, d	7 ± 9.9	8 ± 9.6	0.569
Hospital LOS, d	14 ± 6.5	15 ± 8.5	0.976

Continuous data are expressed as mean ± standard deviation. Categorical data are expressed as the number of patients (percentage)

*Abbreviations*: *ICU* Intensive care unit, *LOS* Length of stay

## Discussion

### Key findings

In adult patients undergoing elective neurosurgery, goal-directed hemodynamic management guided by a non-invasive device was found to be feasible and safe. Key feasibility end points of this study were successfully achieved, the recruitment and consent rates were high, the demographic characteristics in the intervention and control arms were balanced, and no outcome data were missing. No AE, defined as unsatisfactory brain tissue relaxation at the end of surgery or brain edema requiring therapy during surgery or 24 h after surgery, occurred in either arm.

More fluid boluses were administered in the intervention group than the control group, thus suggesting that hemodynamic monitoring identified patients who needed fluid bolus administration more often than standard hemodynamic monitoring. Because colloid solution was the bolus fluid of choice in the intervention group, crystalloids were administered less frequently in the intervention group.

Among efficacy end points, the incidence of postoperative complications, despite being three-time lower in the intervention group, did not reach the threshold for a statistically significant difference. We note that this was a small pilot study aimed primarily at determining feasibility and safety, rather than evaluating efficacy outcomes. The sample size was determined on that basis.

### Comparison with previous studies

Goal-directed hemodynamic management has been investigated in patients undergoing various types of surgical procedures (Corcoran et al. 2012; Grocott et al. 2013; Pearse et al. 2014). GDHT has been associated with a decrease in postoperative morbidity and hospital LOS in recent meta-analyses (Gan et al. 2002; Grocott et al. 2013; Pearse et al. 2014). The main goal of GDHT is the optimization of perioperatively administered fluids (Michard et al., 2012). Administration of an inadequate fluid volume is associated with a higher incidence of postoperative complications (Bellamy et al., 2006), such as hypovolemia (acute renal failure or cerebral ischemia) or hypervolemia (pulmonary or cerebral edema) (Holte et al. 2002). Patients undergoing neurosurgical procedures are at risk of cerebral edema, and fluid restriction is used as a preventive measure but carries a risk of complications associated with hypovolemia. Hemodynamic monitoring can be used to prevent both insufficient and excessive fluid administration. A meta-analysis has indicated that the use of dynamic predictors of fluid responsiveness for hemodynamic therapy positively affects outcomes (Benes et al. 2014; Yang et al., 2014). Stroke volume variation is one such dynamic parameter, which is used to identify patients who positively respond to fluid administration, according to a cutoff value of 10–13% (Yang et al., 2014). In this study, we used a cutoff value of 15% to achieve a balance between excessive fluid restriction and fluid overload. The same strategy and cutoff value have been used in a recent study by Luo (Luo et al. 2017). In our study, a Starling SV monitor was used to measure hemodynamic parameters. This device detects changes in bio-impedance in a completely non-invasive manner and does not require external calibration. The accuracy of bio-impedance-based methods is comparable to that of invasive methods, as reported in a meta-analysis of validation studies (Peyton et al., 2010). The incidence of postoperative complications after neurosurgery varies substantially and has been reported in 9–54% of patients (Manninen et al., 1999; Wong et al. 2012). Several studies have demonstrated a decrease in the rate of postoperative complications when GDHT is used (Lopes et al. 2007; Mayer et al. 2010). The rate of complications in our study was lower in the GDHT group than the control group, although the difference was not statistically significant. However, whether GDHT affected the incidence of postoperative complications could not be determined, because of the small sample size. To demonstrate whether such an effect exists, an RCT with sufficient sample size must be conducted.

### Implications of the study findings

This study supports the hypothesis that a trial of GDHT in adult patient undergoing elective neurosurgery is feasible and safe. The current results may be used for future planning of a fully powered RCT. We suggest omitting the assessment of brain tissue relaxation in a future large trial, because this measure is subjective, and unsatisfactory brain tissue relaxation was not observed in any patients.

### Strengths and limitations

Our study has several limitations. First, this was a single-center trial. Thus, although the results were valid for our institution, anesthesia and neurosurgical practices may vary substantially among other institutions. Second, although the sample size was appropriate to assess safety and feasibility end points, it was insufficient to assess the effects of GDHT compared with standard of care on the incidence of postoperative complications and other secondary outcomes. Finally, the evaluation of brain tissue relaxation as a marker of brain edema, despite having been used in several trials (Li et al. 2016; Dostal et al. 2015), is highly subjective. However, this outcome was dichotomized into favorable and unfavorable, and patients were very unlikely to have been placed in the wrong category, because the difference between categories (sufficiently relaxed vs. stiff) was clear.

## Conclusions

The incidence of postoperative complications is an important patient-oriented outcome. The results of this study indicated the safety and feasibility of a future large trial to examine the effects of goal-directed hemodynamic management on the incidence of postoperative complications in adult patients undergoing elective neurosurgery.

## Supplementary Information

**Additional file 1:** **Supplementary Figure 1.** Hemodynamic management algorithm. MAP, mean arterial pressure; SVV: stroke volume variation; CI: cardiac index.

**Additional file 2:** **Supplementary Table S1.** Definitions of assessed postoperative complications.

## Data Availability

The datasets used and/or analyzed during the current study are available from the corresponding author on reasonable request.

## Abbreviations

AE: Adverse event
AKIN: Acute Kidney Injury Network
ASA: American Society of Anesthesiologists
CI: Cardiac index
CONSORT: Consolidated Standards of Reporting Trials
DVT: Deep vein thrombosis
GDHT: Goal-directed hemodynamic therapy
GIT: Gastrointestinal tract
ICU: Intensive care unit
LOS: Length of stay
MAP: Mean arterial pressure
SVV: Stroke volume variation
