# Supplementary figures and images for: Effects of goal-directed fluid management guided by a non-invasive device on the incidence of postoperative complications in neurosurgery: a pilot and feasibility randomized controlled trial
Source: Perioper Med (Lond). 2023 Jul 5;12:32. doi: 10.1186/s13741-023-00321-3 (PMC10321006; doi:10.1186/s13741-023-00321-3)

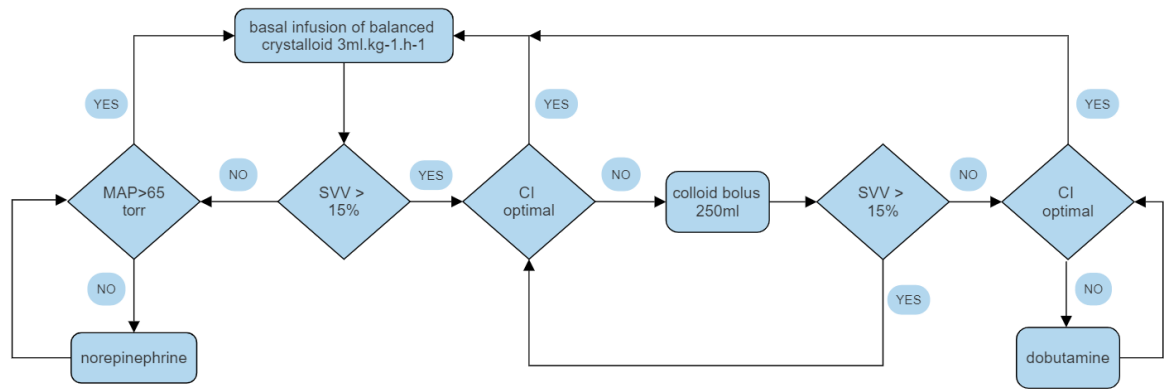

Supplement: Supplementary file 1 — Additional file 1: Supplementary Figure 1. Hemodynamic management algorithm. MAP, mean arterial pressure; SVV: stroke volume variation; CI: cardiac index. [file 13741_2023_321_MOESM1_ESM.pdf]
