# Supplementary material for: Effects of goal-directed fluid management guided by a non-invasive device on the incidence of postoperative complications in neurosurgery: a pilot and feasibility randomized controlled trial
Source: Perioper Med (Lond). 2023 Jul 5;12:32. doi: 10.1186/s13741-023-00321-3 (PMC10321006; doi:10.1186/s13741-023-00321-3)
Supplement: Supplementary file 2 — Additional file 2: Supplementary Table S1. Definitions of assessed postoperative complications. [file 13741_2023_321_MOESM2_ESM.docx]

**Supplementary Table 1. Definitions of Postoperative Complications**

| Cardiovascular | Minor:  Major: | Hypo- or hypertension requiring pharmacotherapy, arrhythmias not requiring pharmacological or non-pharmacological intervention.  Acute myocardial infarction, cardiac failure, arrhythmias requiring pharmacological or non-pharmacological intervention |
| --- | --- | --- |
| Pulmonary | Minor:  Major: | Need for oxygen supplementation therapy.  Need for invasive or non-invasive ventilation, including high-flow nasal oxygenation (in patients extubated in the operating room) |
| Infection | Minor:  Major: | Wound infection, urinary tract infection including asymptomatic bacteriuria, and other non-serious infections requiring systemic antibiotic therapy  Wound infection requiring re-operation or drainage; pneumonia, sepsis |
| Renal | Minor:  Major: | Acute renal failure: AKIN stage I.  Acute renal failure: AKIN stage II or III. |
| Gastrointestinal | Minor:  Major: | Nausea, vomiting or paralytic ileus  GIT bleeding |
| Coagulation | Minor:  Major: | Local bleeding, DVT  Bleeding requiring surgical intervention, pulmonary embolism |
| Neurologic | Minor:  Major: | Cognitive dysfunction or delirium requiring pharmacotherapy  Stroke |

Abbreviations: AKIN, Acute Kidney Injury Network; DVT, deep vein thrombosis

GIT, gastrointestinal.
